# Proteome alterations in the aqueous humor reflect structural and functional phenotypes in patients with advanced normal-tension glaucoma

**DOI:** 10.1038/s41598-022-05273-0

**Published:** 2022-01-24

**Authors:** Si Hyung Lee, Jae Hun Jung, Tae Kwann Park, Chae-Eun Moon, Kyusun Han, Jinhyoung Lee, Hyung Keun Lee, Yong Woo Ji, Chan Yun Kim

**Affiliations:** 1 Department of Ophthalmology, College of Medicine, Soonchunhyang University, Cheonan, Republic of Korea; 2 Department of Ophthalmology, Soonchunhyang University Hospital Bucheon, Bucheon, Republic of Korea; 3 Institute of Vision Research, Department of Ophthalmology, Severance Hospital, Yonsei University, College of Medicine, 50-1 Yonsei-ro, Seodaemun-gu, Seoul, 03722 Republic of Korea; 4 Department of Ophthalmology, Yongin Severance Hospital, Yonsei University, College of Medicine, 363 Dongbaekjukjeon-daero, Giheung-gu, Yongin-si, Gyeonggi-do 16995 Republic of Korea

**Keywords:** Glaucoma, Bioinformatics, Diagnostic markers

## Abstract

Previous reports have shown possible association between altered protein levels in aqueous humor (AH) and normal-tension glaucoma (NTG), but the underlying pathogenetic mechanism as well as specific molecular biomarkers for NTG remains still elusive. Here, we aimed to identify novel biomarkers for advanced NTG by analyzing the proteome of patient-derived AH and their correlation with various functional and structural parameters from the visual field test (VF), optical coherence tomography (OCT), and OCT angiography (OCTA). We determined differentially expressed proteins (DEPs) of the AH of patients with advanced NTG (n = 20) using label-free quantitative (LFQ) proteomics with pooled samples and data-independent acquisition (DIA) analysis with individual samples, and the roles of AH DEPs in biological pathways were evaluated using bioinformatics. We identified 603 proteins in the AH of patients with advanced NTG, and 61 of them were selected as DEPs via global proteome LFQ profiling. Individual DIA analyses identified a total of 12 DEPs as biomarker candidates, seven of which were upregulated, and five were downregulated. Gene ontology enrichment analysis revealed that those DEPs were mainly involved in the immune response. Moreover, IGFBP2, ENO1, C7, B2M, AMBP, DSP, and DCD showed a significant correlation with the mean deviation of VF and with peripapillary and macular parameters from OCT and OCTA. The present study provides possible molecular biomarkers for the diagnosis of advanced NTG.

## Introduction

Glaucoma is a chronic progressive optic neuropathy involving the gradual loss of retinal ganglion cells (RGCs) with characteristic visual-field loss and is a leading cause of irreversible blindness worldwide^1,2^. Primary open-angle glaucoma (POAG) is the most prevalent form of glaucoma, and increased intraocular pressure (IOP) is considered as the most important risk factor for POAG^3^. Normal-tension glaucoma (NTG) is a subset disease entity of POAG with an IOP within the normal range (10–21 mmHg), and is more prevalent in the Asian population^4–6^. In NTG patients, non-IOP factors, such as vascular factors, have been suggested as important causes of glaucomatous damage progression^7–12^, but the exact associations of these factors are still unclear. Although there are cases that progress despite the maintenance of target IOP, the currently available treatments for glaucoma mainly involve interventions to lower the IOP. Therefore, there are still mysteries to unravel regarding this multifactorial disease.

The aqueous humor (AH) is a clear, essential biological fluid that flows from the posterior chamber to the anterior chamber, exiting through the trabecular meshwork. A balance between secretion and drainage of AH is important in the maintenance of IOP^13^. In addition, while circulating inside the eye, AH is enriched with endogenous proteins (normal and pathologic) secreted from nearby intraocular structures. Particularly, AH contains not only proteins secreted from the anterior segment of the eye but also from the posterior segment (including neural tissues) of the eye. A number of studies have reported associations of AH proteome with retinal^14–17^ and neurodegenerative diseases^18,19^.

Previous studies have investigated protein composition differences in the AH between POAG and non-POAG subjects. Early studies used microarray techniques to detect a number of proteins, which were only a subset of the total proteome of AH^20–22^. Recently, as liquid chromatography tandem-mass spectrometry (LC–MS/MS), which provides unbiased information on the total proteome of an AH sample, was introduced, several studies have reported protein composition changes in POAG patients, suggesting potential biomarkers and possible mechanisms involved in glaucoma progression^23–27^. However, there is no previous study regarding AH proteome in patients with NTG. Considering the differences in clinical characteristics between NTG and POAG patients, there is a great need for determining the AH proteome of these patients to elucidate the underlying mechanisms.

In light of the above, in this study, we investigated the composition changes in the AH proteome of patients with advanced NTG. In addition, to investigate the clinical relevance of differentially expressed proteins (DEPs) in AH, we analyzed the association between expression levels of AH DEPs and clinical parameters from the visual field exam (VF), optical coherence tomography (OCT), and OCT angiography (OCTA).

## Results

### Demographic characteristics of subjects

The demographics of subjects enrolled in this study are summarized in Table 1. There were no significant differences between the advanced NTG group and the control group in baseline characteristics, including age and sex. Among the ocular parameters assessed, baseline IOP and axial length were not different between the two groups, while cup/disc ratio was significant larger in the advanced NTG group (*p* < 0.001). Structural and functional parameters from VF, OCT, and OCTA are displayed in Supplementary Table S1.

**Table 1:** Baseline demographic characteristics of normal controls and NTG patients.

Baseline characteristics	Control (n = 20)	NTG (n = 20)	p
Age (range)	67.2 (55–75)	67.2 (60–78)	0.427
Female/male	8 / 12	8 / 12	0.626
Hypertension	5	5	1.000
Diabetes mellitus	5	5	1.000
Hyperlipidemia	3	3	1.000
Cerebrovascular accident	0	0	NA
Cardiovascular disease	2	2	1.000
Baseline IOP	16.75 ± 2.15	16.65 ± 2.08	0.275
Cup/disc ratio	0.35 ± 0.20	0.88 ± 0.10	< 0.001
Axial length	23.36 ± 0.75	23.35 ± 0.65	0.077

*NTG* normal tension glaucoma, *IOP* intraocular pressure.

### Comprehensive profiling of AH proteome in the advanced NTG and control groups

The label-free quantification (LFQ) analysis using pooled sample from each group (n = 5 for each pooled sample) identified a total of 603 proteins at the 1% false discovery rate (FDR) level using the MaxQuant search engine (Supplementary Table S2). To investigate the quantitative reproducibility, we calculated correlation of log2 fold changes between two technical replicates in each biological sample. Based on the results, the duplicate mass spectrometry analysis showed excellent consistency, with an average R^2^ correlation value of 0.953 (Supplementary Fig. S1).

**Figure 1:**
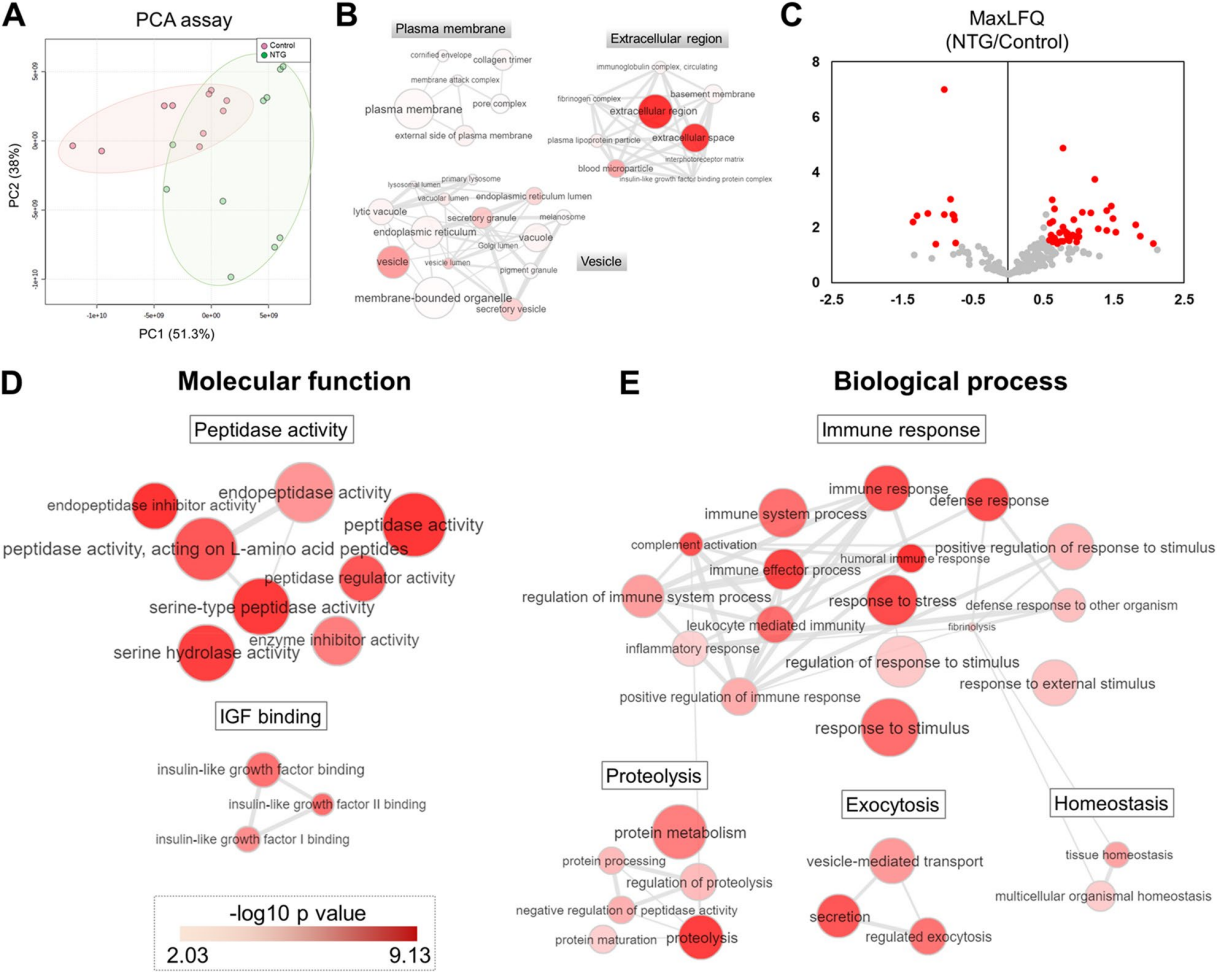
(**A**) Principal component analysis plot for global proteomic data in the control and normal tension glaucoma (NTG) groups. (**B**) Gene Ontology cellular component (GOCC) interactive clusters of proteome in aqueous humor (AH), indicating proteins found in AH were mainly located in plasma membrane, extracellular region, or associated with vesicles. (**C**) Volcano plot displaying the differences in protein expression according to *p* value (y-axis) and the difference in their relative abundance ratio (log2 fold change) in AH between the two groups. Red dots represent differentially expressed proteins (DEPs) with *p* value less than 0.05 and fold change upper than 1.3. (**D**) GO molecular functions significantly enriched by DEPs in NTG compared with the control group are shown. (**E**) Significantly enriched GO biological processes (terms are clustered by representative group (Immune response, Proteolysis, Exocytosis, Homeostasis). GO terms are represented by circles, and semantic similarities were applied to clusters by other GO terms in the gene ontology. GO term is proportional to the size of the circle, whereas the color indicates the − log10 *p* value for the enrichment.

Next, we subjected the obtained proteome profile data to principal component analysis (PCA) analysis to compare the general clustering trends between the NTG and control groups. PCA revealed the clustering of two groups with 51% (PC1), indicating distinct protein expression patterns between the two groups (Fig. 1A). To understand the characteristics of the AH proteome, we investigated the localization of the AH proteome based on the gene ontology (GO) cellular component^28^. Most AH proteins were found to be proteins of the plasma membrane, extracellular region, or associated with vesicles (Fig. 1B).

To identify the AH proteins with altered abundances in NTG, we assessed DEPs in comparison with controls. To avoid endogenously biased comparisons, only 217 proteins with three or more valid values in each group were used for quantification. After the implementation of a normalization process to correct for systematic bias across comparison groups, MaxLFQ based statistical analysis yielded 61 DEPs with an FDR-adjusted *p* value less than 0.05. Among the 61 DEPs, 50 proteins were upregulated and 11 proteins were downregulated in patients with NTG compared with the controls (Fig. 1C; Supplementary Table S3).

### GO biological processes involved in NTG

We examined the biological functions of the 61 DEPs by performing enrichment analysis of GO and Reactome pathway (Supplementary Table S4). The DEPs were significantly (*p* < 0.01) involved in the molecular functions related to peptidase activity and insulin-growth Factor (IGF) binding (Fig. 1D). Furthermore, GO biological process analysis revealed that the DEPs in NTG were mainly involved in processes related to the immune response, proteolysis, exocytosis, and homeostasis (Fig. 1E). The IGF binding and immune response pathways were also identified by Reactome pathway analysis.

### Data independent acquisition (DIA) analysis for verification of DEPs

**Figure 2:**
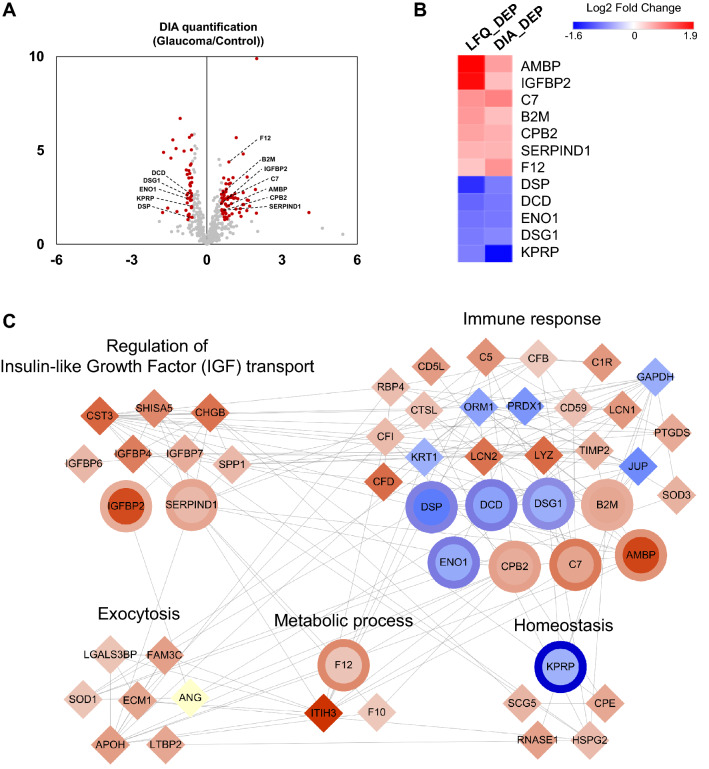
(**A**) Volcano plot depicting the variance in expression of proteins in the aqueous humor (AH) between the normal tension glaucoma (NTG) and control groups. Red dots represent significantly differentially expressed proteins (DEPs) (fold change > 1.5, *p* value < 0.05). Twelve DEPs in the label free quantification (LFQ) and data independent acquisition (DIA) analysis were consistently highlighted and labeled. (**B)** Heatmap showing the relative abundance of 12 DEPs consistently altered in the LFQ and DIA analysis. (**C)** Protein–protein interaction Network of AH DEPs in the NTG group versus the control group. Network model showing the biological processes affected, including the immune response, insulin-like growth factor transport, exocytosis, metabolic processes, and homeostasis. The colors of the nodes represent proteins whose levels were greatly increased (red) or decreased (blue) in NTG. The gray lines between nodes represent either a regulatory role or a physical interaction between proteins. Circle shaped nodes represent 12 verified DEPs (inner node: abundance change in the LFQ, outer layer: abundance change in DIA).

To verify altered protein expression, we performed DIA analysis using 10 individual AH samples in each group. As a result, 68 proteins were significantly up-regulated and 43 proteins were down-regulated with a fold change > 1.5 in relative abundance and a *p* value < 0.05 (Fig. 2A; Supplementary Table S5). Interestingly, a total of 12 DEPs verified by DIA analysis showed consistent increase and decrease trends compared with the LFQ analysis. As shown in Fig. 2B, the heat map revealed that 7 proteins simultaneously increased (AMBP, alpha-1-microglobulin/bikunin precursor; IGFBP2, IGF binding protein 2; C7, complement component 7; B2M, beta-2-microglobulin; CPB2, carboxypeptidase B2; SERPIND1, heparin cofactor 2; F12, coagulation factor XII) and 5 proteins decreased (DSP, desmoplakin; DCD, dermcidin; ENO1, enolase 1; DSG1, desmoglein-1; KPRP, keratinocyte proline-rich protein) in LFQ and DIA, respectively.

To explore the systematic functions of the aforementioned NTG-associated processes, we constructed a protein–protein interaction network describing the biological connection among DEPs (Fig. 2C). The network model demonstrated a significant activation of IGF transport (CST3, SHISA5, CHGB, IGFBP6, IGFBP4, IGFBP7, SPP1, IGFBP2, SERPIND1), exocytosis (LGALS3BP, FAM3C, SOD1, ECM1, APOH, LTBP2), and metabolic processes (ITH3, F10, F12). By contrast, the proteins in immune response and homeostasis processes were largely up-regulated in NTG, while conversely, a few proteins were down-regulated, indicating complex proteome changes in those process in the AH of NTG patients. Notably, 12 DEPs altered in LFQ and DIA belonged to IGF transport (IGFBP2, SERPIND1), metabolic process (F12), homeostasis (KPRP), and immune response (DSP, DCD, DSG1, ENO1, B2M, CPB2, C7, AMBP).

### Correlation between clinical parameters and protein expression levels

To evaluate the clinical significance of the DEPs detected in the AH of advanced NTG patients, we further investigated the correlation between clinically important parameters from VF, OCT, and OCTA and expression levels of DEPs. As shown in Table 2, a total of 8 of 12 DEPs were significantly correlated with the indices of VF, OCT, and OCTA. IGFBP2 showed a significant negative correlation with the mean deviation (MD) from the VF test, while no AH protein showed significant correlation with visual field index. An evaluation of the OCT parameters showed that none of the DEPs was significantly correlated with average retinal nerve fiber layer (RNFL) thickness, whereas ENO1 and DCD levels showed a positive correlation (and C7 and B2M levels showed a negative correlation) with average ganglion cell-inner plexiform layer (GCIPL) thickness.

**Table 2:** Correlation between clinical parameters and expression levels of AH proteins in patients with NTG.

Clinical parameters	Protein (Gene name)	r^a^	*p*^*b*^
Baseline IOP	–	–	–
IOP fluctuation	–	–	–
**VF**			
MD	IGFBP2	− 0.758	0.011
VFI	–	–	–
**OCT parameters**			
Mean RNFL thickness	–	–	–
Mean GCIPL thickness	ENO1	0.729	0.017
	C7	− 0.693	0.026
	B2M	− 0.669	0.035
	DCD	0.687	0.028
**OCTA parameters**			
Optic disc			
Perfusion	KPRP	0.770	0.009
Flux index	KPRP	0.673	0.033
Macula			
Vascular density	ENO1	0.697	0.025
	C7	− 0.842	0.002
	DSP	0.685	0.029
	B2M	− 0.721	0.019
	DCD	0.879	0.001
Perfusion density	AMBP	− 0.665	0.036
	ENO1	0.652	0.041
	C7	− 0.860	0.001
	DSP	0.646	0.043
	B2M	− 0.726	0.018
	DCD	0.848	0.002
Microvasculature dropout	DCD	− 0.661	0.037

^a^Spearman coefficients (r), ^b^*p *values.

*AH* aqueous humor, *AMBP* alpha-1-Microglobulin/Bikunin Precursor, *B2M* beta-2-microglobulin, *C7* complement component 7, *DCD* dermcidin, *DSP* desmoplakin, *ENO1* alpha-enolase, *GCIPL* ganglion cell inner plexiform layer, *IGFBP2* insulin growth factor binding protein 2, *IOP* intraocular pressure, *KPRP* keratinocyte proline-rich protein, *MD* mean deviation, *OCT* optical coherence tomography, *OCTA* OCT angiography, *RNFL* retinal nerve fiber layer, *VF* visual field, *VFI* visual field index.

**Figure 3:**
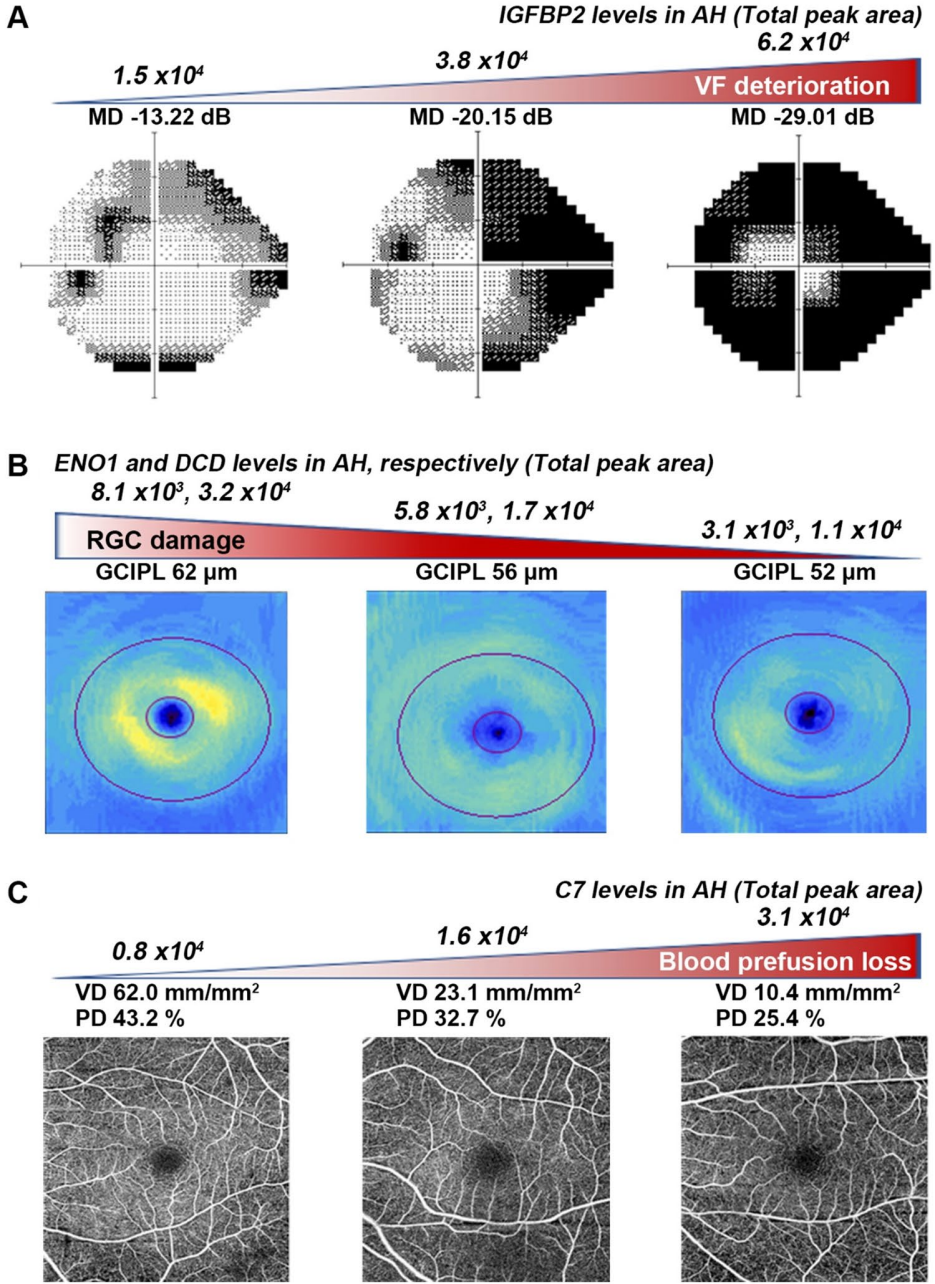
Representative cases showing the correlation between visual field (VF), optical coherence tomography (OCT) and OCT angiography (OCTA) parameters, and protein levels of aqueous humor (AH) in patients with normal tension glaucoma. (**A)** Insulin-growth factor binding protein 2 (IGFBP2) in AH showed a significant correlation with the mean deviation (MD) value from the VF test. (**B)** Alpha-enolase (ENO1) and dermcidin (DCD) levels in AH were significantly associated with a decrease in macular ganglion cell inner plexiform layer (GCIPL) thickness representing retinal ganglion cell (RGC) damage. (**C)** Reduced retinal vascular (VD) and perfusion density (PD). Blood perfusion was significantly related to AH protein expression of complement component 7 (C7).

Among the OCTA parameters, KPRP was the only AH protein that was positively and significantly correlated with both optic disc perfusion and flux index, while a number of proteins, including AMBP, ENO1, C7, DSP, B2M, and DCD showed significant correlations, either positive or negative, with macular vascular density or perfusion density. Furthermore, the DCD expression level showed a significant negative correlation with the presence of peripapillary choroidal microvasculature dropout (MvD). No AH protein showed a significant correlation with baseline IOP and IOP fluctuation of the enrolled NTG patients. Representative cases are shown in Fig. 3 to illustrate the correlation between the expression levels of selected proteins—including IGFBP2, ENO1, DCD, and C7—and parameters from VF, macular OCT, and OCTA.

**Figure 4:**
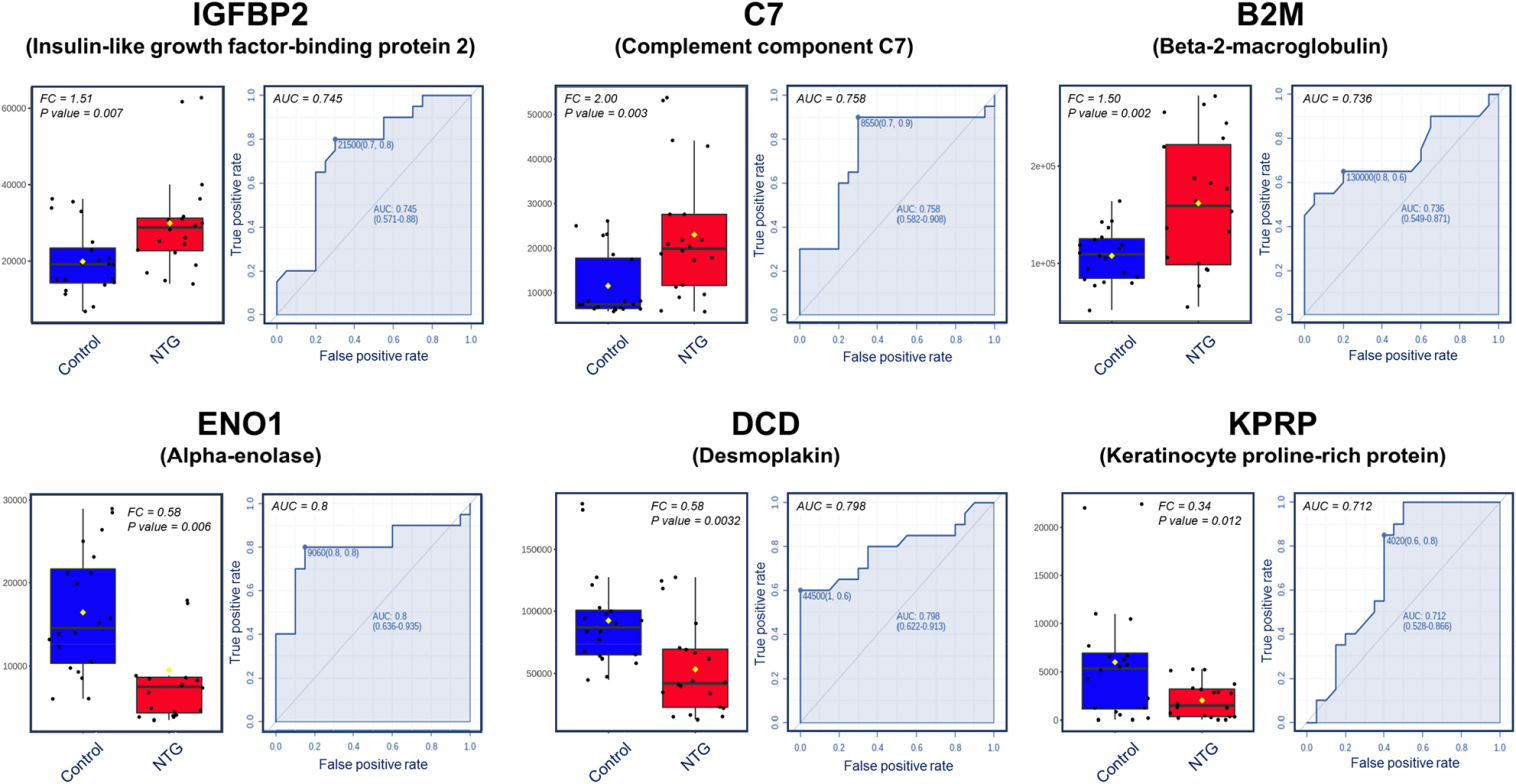
Box plot and receiver operating characteristic (ROC) curve of six selected marker proteins in the aqueous humor (AH) of patients with normal tension glaucoma (NTG). The box plot demonstrates the differences of protein abundance in AH between the control and NTG groups. The fold change (FC) and *p* value for each marker are indicated on the interactive box plots. The ROC curve yielded an area under the curve (AUC) that indicates diagnostic efficiency.

The diagnostic potential of the six proteins was further evaluated by receiver operating characteristic (ROC) analysis. Figure 4 depicts the sensitivity and specificity for six proteins related to NTG (IGFPB2, C7, B2M, ENO1, DCD, and KPRP) with area under the curve (AUC) values higher than 0.7 indicating a remarkable ability of the classifier to distinguish NTG patients from normal controls.

**Figure 5:**
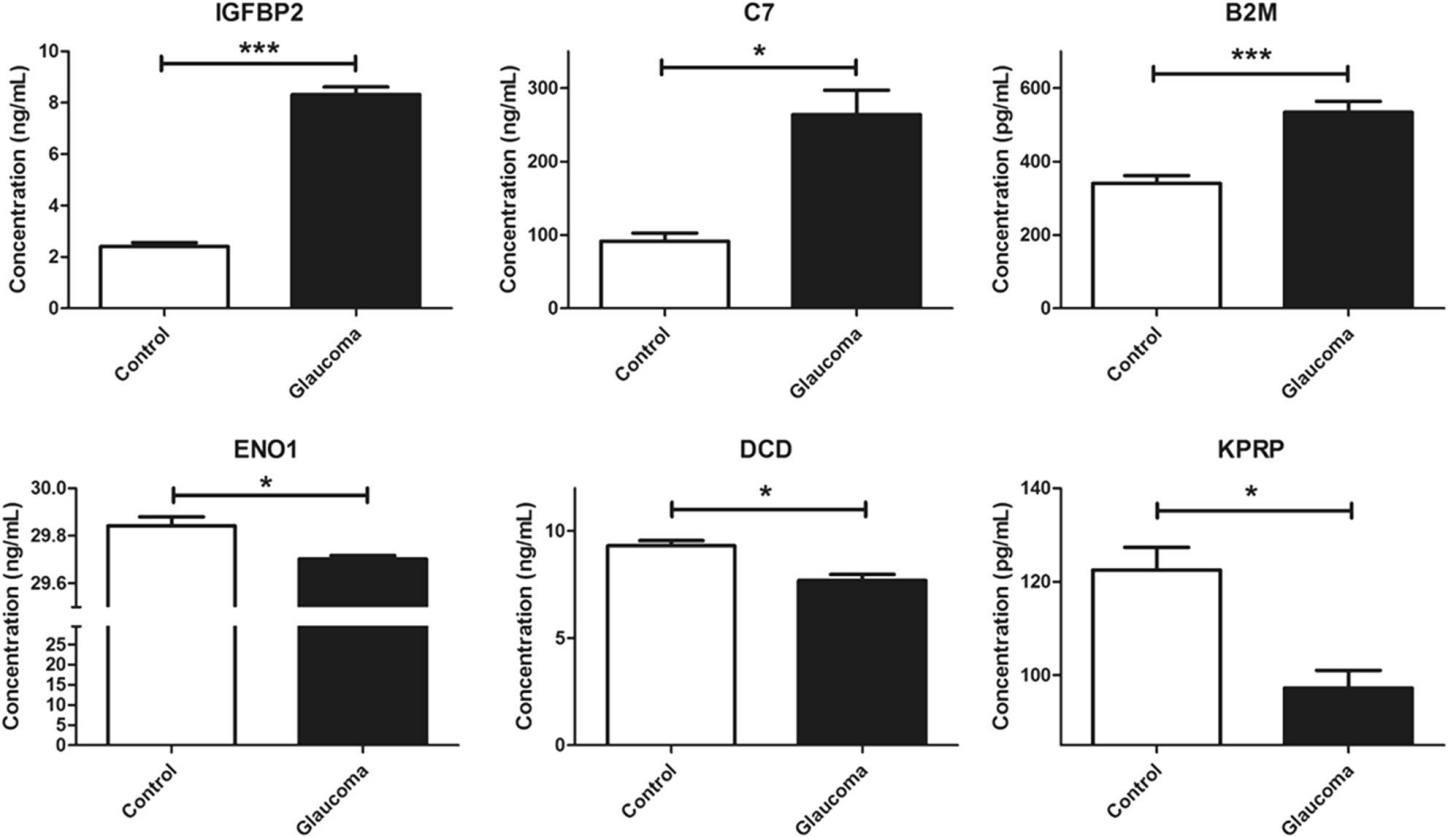
Measurement of six selected marker protein levels using ELISA. Bar graphs demonstrate expression levels of six marker proteins, including IGFBP2, C7, B2M, ENO1, DCD, and KPRP. All experiments were repeated three times in at least triplicate (n = 5, Mann–Whitney U test for statistical analysis). *p < 0.05, **p < 0.01, ***p < 0.001.

To further validate the six proteins which showed significant correlation with VF, OCT, and OCTA, we conducted ELISA and compared the concentration of six proteins between the control and NTG subjects (n = 5 from each group). As shown in Fig. 5, expression of IGFPB2, C7, and B2M was significantly upregulated in AH of NTG subjects compared to that of normal control (p < 0.001 for IGFBP2 and B2M, p < 0.05 for C7), while expression of ENO1, DCD, and KPRP was significantly downregulated in AH of NTG group compared to the controls (p < 0.05 for all three proteins).

## Discussion

A few studies have been reported regarding proteome changes in POAG compared with non-glaucomatous controls^23–27^. While the main pathogenesis of POAG involves increased IOP level, studies have focused on on the functional changes of trabecular meshwork outflow facility by looking at protein expression changes in the AH of POAG patients. However, in NTG, which is not associated with increased IOP, the proteomic changes found in the AH may indirectly reflect pathogenetic changes in RGCs or in the optic nerve head. In this study, we applied two sequential approaches using high-resolution LC–MS/MS, which are mainly employed in biomarker research: MaxLFQ for global quantitative proteomics and DIA quantification for accurate verification. Among 603 identified proteins from AH with NTG, 51 DEPs were observed in global profiling analysis, and 12 DEPs were finally identified in a DIA analysis, which may be considered as candidates for AH biomarkers for NTG. These AH biomarker candidates for NTG were found to be mainly involved in the immune response as well as in IGF transport regulation, exocytosis, metabolic processes, and homeostasis. In particular, the expression levels of 8 DEPs, namely IGFBP2, ENO1, C7, B2M, DCD, KPRP, DSP, and AMBP, were found to be significantly associated with changes in VF, OCT, and OCTA, which reveals the clinical relevance of AH proteins in NTG.

There have been a number of evidences suggesting neuroinflammation is one of a key pathogenetic mechanism underlying glaucoma damage and development^29,30^. A number of molecular pathways have been suggested as major regulators of neuroinflammation that may be implicated in glaucoma pathogenesis, including the complement cascade^31–35^, Toll-like signaling^36,37^, and tumor necrosis factor α pathway^38–42^. Moreover, various inflammatory cytokines, interferons, interleukins, and proteins involved in antigen presentation to T cells have previously been reported to be involved in glaucoma^43–45^. Recently, several studies reported that proteins related to immune system were highly abundant in AH of POAG patients compared to control^24,25,46^. Consistent with previous findings, our DEPs in AH in patients with NTG were involved in immune response, including B2M, CPB2, and C7 as commonly identified proteins from previous POAG studies. Specifically, B2M (β2-microtubulin), is a well-known major histocompatibility complex class I molecule involved in antigen presentation, and C7 is reported to be involved in the innate immune response. AMBP is a complex glycoprotein reported to play a role in the regulation of inflammatory processes. From these findings, we may speculate the proteome changes observed in AH of glaucoma patients may indicate neuroinflammation may contribute to glaucoma development and progression. However, further evidences are needed to reveal whether our results are true reflection of inflammatory process occurring at the optic nerve head and retina, which is located at the posterior pole of eyeball.

While previous studies have focused on proteome changes in glaucoma patients, we further sought for any correlation between DEPs and various parameters from VF, OCT, and OCTA. In this study, the expression level of IGFBP2 was significantly correlated with MD values. Previously, the *IGFBP2* gene was reported to be significantly associated with optic disc morphology in the Dutch population^47^, indicating a possible relationship with glaucoma severity. Among OCT and OCTA parameters, several proteins were associated with macular GCIPL thickness as well as macular vascular and perfusion density, which include ENO1, C7, B2M, AMBP, DSP, and DCD. Especially, DCD showed a significant negative correlation with peripapillary choroidal MvD. While C7, B2M, and AMBP are mainly involved in immune responses, ENO1 is reported to be involved in glycolytic process and its antibodies were mostly expressed in retinal ganglion cells and inner nuclear cells^48^. DSP is a junctional protein required to maintain epithelial and vascular tissue integrity and has been previously reported to be increased in the peripapillary sclera in an experimental glaucoma model^49^. While not much is known about DCD, an anti-microbial peptide involved in proteolysis, it was reported to be present in the AH of rabbit eyes^50^, and was previously recognized as a survival-promoting peptide in several cell lines under hypoxic or oxidative stress^51,52^.

Interestingly, in the peripapillary OCT and OCTA analysis, only KPRP showed a significant association with OCTA parameters. We speculate that the reason for such finding was that the NTG patients were advanced cases with an RNFL thickness reaching a floor effect in the OCT and OCTA measurements, which is not the case of macular parameters, according to previous reports^53–56^. Taking this into account, the DEPs from the AH of the advanced NTG patients included in this study may be potential biomarkers for the diagnosis of advanced NTG cases and the confirmation of the NTG stage. However, further studies including large cohorts at each glaucoma stage should be conducted to evaluate the relationship between AH biomarker proteins and disease severity.

There are several limitations in this study. First, the limited number of subjects may weaken the clinical implications of our study. However, we carefully matched the groups for demographic characteristics, and no significant differences were found for other possible confounding factors including various systemic vascular comorbidities. Nevertheless, the findings of the present study should be validated in larger samples. Second, we did not consider the effects of IOP lowering eyedrops on AH protein composition, including prostaglandin analogue (PGA), beta-blocker, alpha-agonist, and carbonic anhydrase inhibitor agents. We included advanced NTG patients who were on maximal tolerated medical therapy to minimize the effects of anti-glaucoma eyedrops on AH proteome expression among NTG patients, but there is the possibility that eyedrop usage might affect the protein composition of AH. However, there have been no reports regarding the possible effects of anti-glaucoma agents on AH protein expressions, and further research is needed to reveal the effects of IOP lowering agents on AH proteome. Third, careful interpretation is needed when looking at the results of this study since AH proteome changes may not directly reflect structural and functional changes occurring in the optic nerve head and retina. However, there is continuous exchange between AH and vitreous humor located in the posterior part of the eye, and we may expect that a large composition of proteins found in vitreous may be similar to those found in AH^57–59^. Further studies are needed to confirm the findings from this study that AH proteome alteration may have implications on underlying pathogenesis of NTG.

In summary, this study demonstrated the changes in AH proteome composition in patients with advanced NTG, and a large portion of DEPs found in AH of NTG patients were involved in immune responses. The significant correlation between AH DEPs and clinically essential parameters indicates that AH proteins may be considered as biomarker candidates for advanced NTG, which should be further evaluated in future studies.

## Methods

### Subject enrollment and aqueous humor sampling

This cross-sectional case–control study was conducted at Soonchunhyang University Hospital Bucheon. We enrolled a total of 40 subjects: 20 NTG patients and 20 age/sex-matched control subjects with simple cataract formation. Optic disc examination was performed to screen any glaucomatous optic disc changes using slit-lamp examination and optic disc photographs (Zeiss Clarus 500 fundus camera; Carl Zeiss Meditec, Dublin, CA, USA), and VF test was performed using Humphrey visual field analyzer (Carl Zeiss Meditec). OCT (RNFL and GCIPL thickness) and OCTA parameters (optic disc perfusion/flux index and macular vascular/perfusion density) were obtained with Cirrus HD-OCT (Carl Zeiss Meditec). Peripapillary choroidal MvD was defined as a focal capillary dropout with no detectable microvascular network located at the peripapillary area in the deep-layer en-face images, and was suspected to include the entire choroidal thickness. Axial length was measured with an IOL Master (Carl Zeiss Meditec).

The inclusion criteria for NTG group were deep anterior chamber depth (> 4 central corneal thickness) and open angle on slit-lamp examinations. Clinical diagnosis of NTG was made based on IOP measurement, optic disc examination, VF test, spectral-domain OCT results. Baseline IOP was recorded using averaged IOP from two IOP readings measured by one glaucoma specialists (SHL) during office hours, and IOP fluctuation was defined as standard deviation of IOP during last 2 year follow-up period before the surgery. Glaucomatous damage was defined as RNFL defects or glaucomatous optic disc changes with corresponding VF defects, confirmed by at least 2 reliable VF examinations. Only reliable VF test results with false-positive errors < 15%, false-negative errors < 15%, and fixation loss < 20% were included in the study, and patients with MD < 12 dB on the standard Swedish interactive threshold algorithm 24–2 program were included in the current study. All NTG subjects were on maximally tolerated medical therapy, using eyedrops containing prostaglandin analogues, beta-blocker, alpha-agonist, and carbonic anhydrase inhibitor. For both control and NTG group, subjects with baseline IOP exceeding 21 mmHg or with any previous history of ophthalmic surgery were excluded, and also subjects with concomitant other ophthalmic or neurologic disease that may possibly affect VF testing results were excluded.

We collected AH from 40 subjects (20 control, 20 NTG subjects) undergoing either cataract surgery or glaucoma surgery (filtration or aqueous tube shunt surgery). The AH was sampled from subject’s eye by puncturing the cornea with 30 gauge needle before performing any surgical steps, with amount of 0.5–1.0 mL of AH. During this procedure, intraocular tissues including iris and lens were not violated. The AH samples were then transferred to Biobank in Soonchunhyang University Hospital Bucheon and stored in a deep freezer at − 80 °C for future proteomic analysis. This study was approved by the institutional review board of the Soonchunhyang University Hospital Bucheon (IRB No. 2020-07-011-001), informed consent was obtained from all subjects for AH sampling and analysis. All procedures performed in the present study adhered to the tenets of the Declaration of Helsinki.

### Sample preparation for proteomic analysis

Five AH samples from control and NTG subjects were used for global profiling with LFQ. For verification, an additional ten AH samples from each group were subjected to DIA analysis (Supplementary Fig. S2). For each AH protein sample, we performed depletion of highly abundant proteins using Seppro IgY spin columns (Sigma Aldrich, St. Louis, MO, USA) to facilitate the detection of potential marker proteins presented at low abundance in AH. The protein concentration was measured using the BCA protein assay, following the manufacturer’s instructions (Thermo Scientific, Rockford, IL). Samples of 100 μg total protein were digested into peptides by in-solution digestion as described previously^60^. Briefly, 10 M urea in 100 mM ammonium bicarbonate was mixed with each sample (v/v, 1:1) and the mixture was incubated for 30 min at room temperature for denaturation. Reduction and alkylation of the cysteine residues were then performed with 10 mM dithiothreitol and 30 mM iodoacetamide, respectively. The samples were then digested with trypsin at a 50:1 (w/w) protein-to-protease ratio and incubated overnight at 37 °C. The activated trypsin reaction was terminated with 0.4% trifluoroacetic acid, and peptides were desalted with a C18 spin column (Thermo, Rockford, IL, USA). The resultant peptides were dried and stored at − 80 °C. A retention time normalization kit (iRT peptides, Biognosys, Switzerland) was used to spike samples at a concentration of 1:20 v/v in all samples as an external standard.

### Quantitative global profiling

All samples were processed individually for LC–MS/MS. Digested AH samples were re-suspended in 0.1% formic acid in water and analyzed using the Q Exactive orbitrap plus hybrid mass spectrometer (Thermo Fisher Scientific, CA, USA) coupled with the EASY-nLC 1000 system (Thermo Scientific, Bremen, Germany). For the proteome profiling analysis and DIA verification, peptide samples were separated on an easy spray column (2 μm C18 particles, 50 cm × 75 μm ID), with a 120 min gradient (from 5 to 35% solvent B over 90 min, from 35 to 50% solvent B over 5 min, 80% solvent B for 10 min, and 5% solvent B for 10 min) and analyzed by mass spectrometry. Solvents A and B were 0.1% formic acid in water and 0.1% formic acid in acetonitrile, respectively. The peptides were loaded onto a trap column (75 μm × 2 cm, 3 μm, C18, 100 Å). Eluted peptides were ionized through an EASY-spray column (50 cm × 75 μm ID) packed with 2-μm C18 particles at an electric potential of 2.0 kV. The column temperature is maintained at 60 °C using a column heater. Full MS survey scans were acquired in a scan range of 350–2,000 Th with a resolution of 70,000 at m/z 200. A data-dependent top 10 method was operated during which higher-energy collisional dissociation (HCD) spectra were obtained at 17,500 MS2 resolution with automated gain control (AGC) target of 1 × 106 and maximum ion injection time (IT) of 50 ms. The top ten abundant ions with an isolation window of 2.0 m/z were selected and fragmented by data-dependent MS/MS experiments and exclusion duration of 30 s and at a normalized collision energy of 27 for HCD. The charge state of 1 was discarded. Maximum ion injection times for full MS survey scans and MS/MS scans were 100 ms and 50 ms, respectively. The AGC target value was set to 1.0 × 106 for both MS and MS/MS scans.

### Database search and quantitative analysis

The MS2 spectra were processed using the MaxQuant (v. 1.5.7.4, Max-Planck-Institute of Biochemistry, Munich, Germany) against the Uniprot human database^61^. MS/MS searches performed with the following parameters: fixed modification of Carbamidomethylation, *N*-acetylation of protein, and variable oxidation of methionine. The required FDR of 1% was applied at the peptide spectrum match (PSM), protein levels and modification level. An initial precursor was matched to 4.5 ppm tolerance and a 20 ppm for fragment spectra. Proteins were analyzed using the XIC-based LFQ algorithm in MaxQuant (Max-Planck-Institute of Biochemistry). To maximize the number of quantification across samples, the retention times of all quantified samples were aligned using “match between runs” option. The match time window was 0.7 min, and the alignment time window was 20 min. Before loading LFQ intensity data, we processed to eliminate reverse database, contaminants, and proteins only identified by site. All LFQ intensities were processed using a log2 scale. Proteins that did not indicate all values in at least one group were filtered out. Additionally, missing values were imputed by normal distribution (using a width of 0.3 and a downshift of 1.8). Using LFQ intensities, proteins with expression greater than ± 1.3 (for global profiling) and 1.5 (for DIA analysis) fold change from Student’s t test were considered as DEPs, and further enrichment analyses were conducted using DEPs.

### Individual DIA analysis and data processing

For DIA analysis, a retention time normalization kit (iRT peptides, Biognosys, Switzerland) was used to spike samples at a concentration of 1:20 v/v in all samples as an external standard^62^. 2 μg of each peptide sample was analyzed using Q-Exactive plus (Thermo Fisher Scientific) equipped with an EASY-nLC 1000 UHPLC System (Thermo Fisher Scientific). DIA method covers the mass range from 500 to 900 m/z with a resolution of 170,000 at 200 m/z. The AGC target was set at 1e6 with a 60-ms maximum injection time. The normalized collision energy for HCD-MS2 experiments was set to 30%, the AGC target was set at 2 e5, and the maximum injection time was set to 60 ms.

The DIA data were analyzed with Spectronaut Pulsar (version 11.0.15038.4.29119, Biognosys) using a search archive spectral library, and the default settings were used for targeted analysis. In brief, a dynamic window for the XIC extraction window and a non-linear iRT calibration strategy were used. Mass calibration was set to local mass calibration. Interference correction on the MS1 and MS2 levels was activated, eliminating fragments/isotopes from quantification based on the presence of interfering signals. The FDR was set to 1% at the peptide precursor level and 1% at the protein level.

### Enrichment analysis using GO and protein–protein interaction network

The GO of proteins was classified using g:Profiler (https://biit.cs.ut.ee/gprofiler)^63^ to explore the functionality of altered GO biological process and GO molecular function in AH associated with glaucoma. A cutoff of p value < 0.05 was adjusted for all GO categories. GO enrichment analysis results were reduced and summarized by semantic similarity using REVIGO (http://revigo.irb.hr/) which is web-based tool^64^. To construct the protein–protein interaction networks, we interrogated protein–protein interactome information from the STRING version 11 public database (https://string-db.org/). The network model was visualized using Cytoscape (v.3.8.2, National Institute of General Medical Sciences, Bethesda, USA). Other statistical analysis including the box plot and the corresponding ROC curve have been generated using Metaboanalyst 4.0 server (https://www.metaboanalyst.ca/).

### ELISA

To further confirm the expression level of DEPs, commercialized ELISA was performed to measure the concentration of DEPs including IGFBP2 (MBS177374, MyBioSource, CA, USA), ENO1 (MBS706020, MyBioSource), DCD (EK13611, Signalway Antibody, MD, USA), C7 (KA2115, Abnova, Taipei City, Taiwan), KPRP (MBS761698, MyBioSource), and B2M (ab99977, Abcam, Cambridge, UK). Five AH samples from each group were used for ELISA. All experiments were repeated three times in at least triplicate.

### Statistical analysis

Statistical significance for both demographic characteristics and DEPs was determined using Student’s t test or Fisher’s exact test, ROC analysis was conducted to determine diagnostic potentials for NTG diagnosis using selected DEPs. Correlation between mean protein levels and various clinical tests parameters were performed using Spearman correlation test. Statistical analyses were performed using SPSS version 26.0 (IBM Corp., Armonk, NY, USA), and differences at p value less than 0.05 were considered statistically significant.

## Supplementary Information

Supplementary Information 1.

Supplementary Information 2.

Supplementary Information 3.

Supplementary Information 4.

Supplementary Information 5.

Supplementary Information 6.

Supplementary Information 7.
