# Supplementary material for: Proteome alterations in the aqueous humor reflect structural and functional phenotypes in patients with advanced normal-tension glaucoma
Source: Sci Rep. 2022 Jan 24;12:1221. doi: 10.1038/s41598-022-05273-0 (PMC8786875; doi:10.1038/s41598-022-05273-0)
Supplement: Supplementary file 1 — Supplementary Information 1. [file 41598_2022_5273_MOESM1_ESM.docx]

**
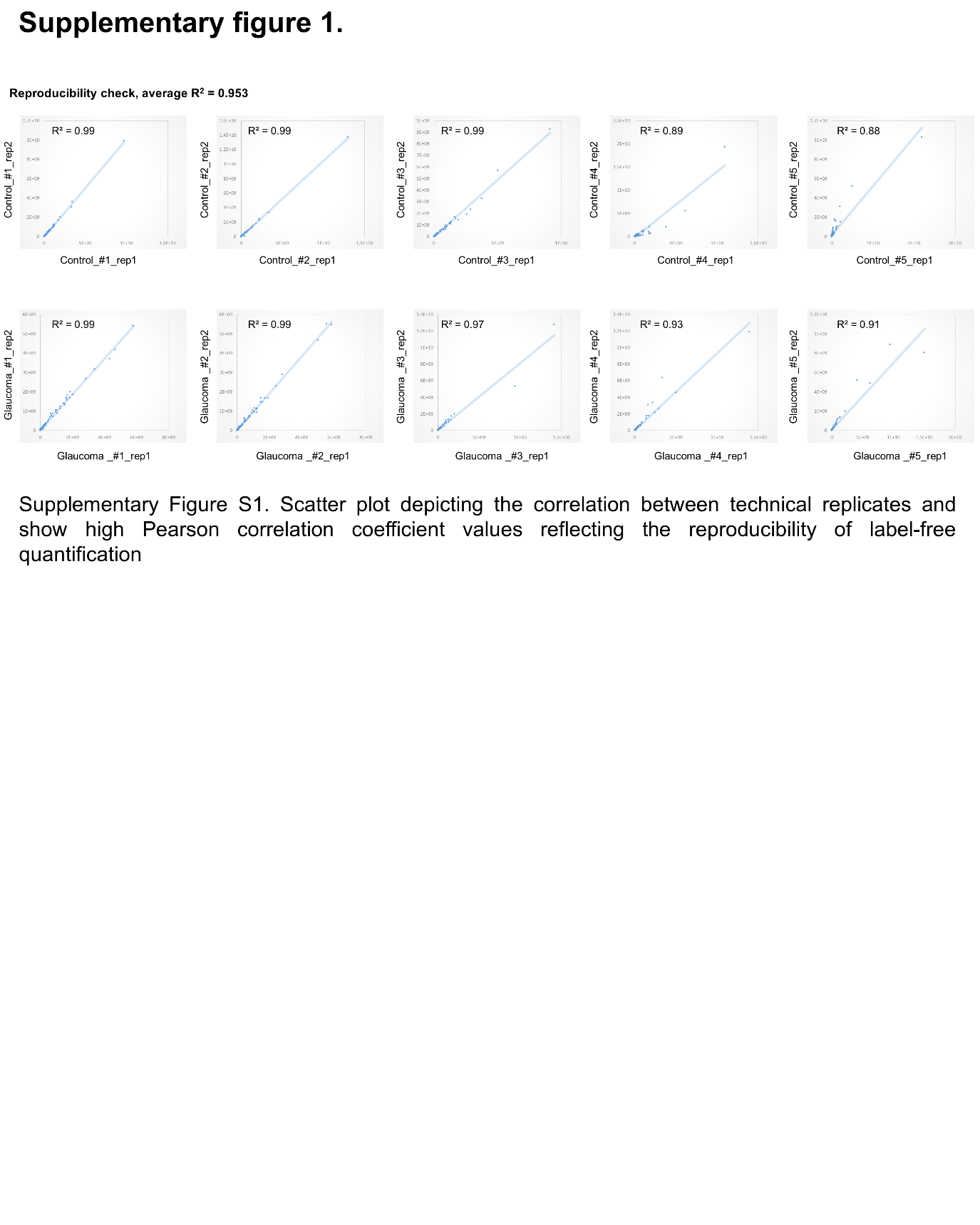
**

**Supplementary figure 2. Scatter plot depicting the correlation between technical replicates and show high Pearson correlation coefficient values reflecting the reproducibility of label-free quantification**
