# Supplementary material for: Proteome alterations in the aqueous humor reflect structural and functional phenotypes in patients with advanced normal-tension glaucoma
Source: Sci Rep. 2022 Jan 24;12:1221. doi: 10.1038/s41598-022-05273-0 (PMC8786875; doi:10.1038/s41598-022-05273-0)
Supplement: Supplementary file 2 — Supplementary Information 2. [file 41598_2022_5273_MOESM2_ESM.docx]

**
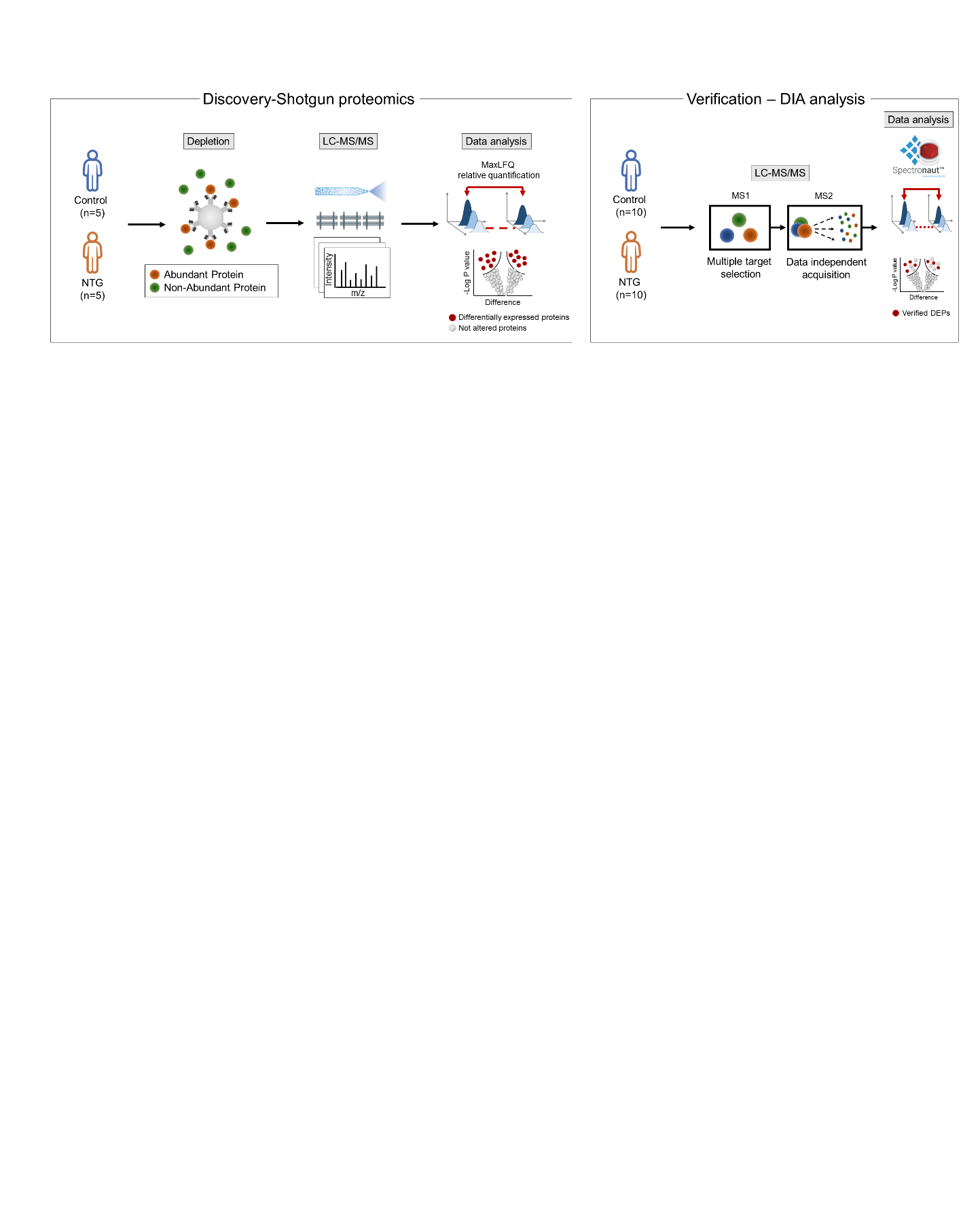
**

**Supplementary figure 1. Schematic representation of the experimental workflow for label free quantification (LFQ) proteomic analysis (left panel) and data independent acquisition (DIA) verification (right panel) using liquid chromatography tandem-mass spectrometry (LC-MS/MS).**
